# TyG Index and Frailty as Composite Biomarkers of Cardiometabolic Risk and Mortality Across CKM Stages 0–3

**DOI:** 10.3390/metabo16060426

**Published:** 2026-06-17

**Authors:** Yaocheng Luo, Peng Zeng, Shuoya Huang, Zhenzhen Peng, Jian Zheng, Zumin Shi, Manoj Sharma, Yong Zhao

**Affiliations:** 1 School of Public Health, Chongqing Medical University, Chongqing 400016, China; 2024111689@stu.cqmu.edu.cn (Y.L.); 2025121935@stu.cqmu.edu.cn (P.Z.); 2025121883@stu.cqmu.edu.cn (S.H.); 2025121908@stu.cqmu.edu.cn (Z.P.); 2025121943@stu.cqmu.edu.cn (J.Z.); 2 Research Center for Medicine and Social Development, Chongqing Medical University, Chongqing 400016, China; 3 Research Center for Public Health Security, Chongqing Medical University, Chongqing 400016, China; 4 Nutrition Innovation Platform-Sichuan and Chongqing, School of Public Health, Chongqing Medical University, Chongqing 400016, China; 5 Human Nutrition Department, College of Health Sciences, QU Health, Qatar University, Doha 2713, Qatar; zumin@qu.edu.qa; 6 Department of Social and Behavioral Health, School of Public Health, University of Nevada, Las Vegas, NV 89106, USA; manoj.sharma@unlv.edu; 7 Department of Internal Medicine, Kirk Kerkorian School of Medicine, University of Nevada, Las Vegas, NV 89106, USA

**Keywords:** CKM syndrome, triglyceride-glucose index, frailty index, cardiovascular disease, mortality

## Abstract

**Background**: Cardiovascular disease and mortality are common outcomes of cardiovascular–kidney–metabolic (CKM) syndrome. The integrated role of metabolic dysfunction and frailty, quantified by the triglyceride–glucose–frailty index (TyG-FI), remains insufficiently explored. This study examined the association between TyG-FI and incident composite outcomes among participants with CKM stages 0–3. **Methods**: Data were obtained from two large cohort studies conducted in China and the United States. The analysis focused on participants classified as CKM stages 0–3. Cox proportional hazards models were used to estimate the relationship between TyG-FI and incident composite outcomes. Nonlinear associations were explored using spline functions. Additional analyses were performed across different subgroups and under varied assumptions. Model performance over time was also assessed. **Results**: Significant differences in outcome incidence were observed across TyG-FI levels. Higher quartiles showed a gradual increase in risk and displayed a dose–response pattern, with inflection points at 1.01 and 2.29. Associations were consistent across subgroups, and TyG-FI demonstrated moderate discrimination (AUCs 0.714 and 0.744). **Conclusions**: In the CHARLS and HRS cohorts, higher TyG-FI scores were independently associated with an increased risk of incident composite outcomes among participants with CKM stages 0–3, with a nonlinear relationship observed. Its discriminatory power was moderate, suggesting that TyG-FI may serve as a supplementary indicator for risk stratification in the early to mid-stages, although its clinical predictive value requires further validation.

## 1. Introduction

Findings from the 2023 Global Burden of Disease assessment indicate a substantial increase in the global burden of cardiovascular disease over time, with total cases rising from 271 million in 1990 to 626 million in 2023, and around 19 million deaths each year [1,2]. Cardiovascular Disease (CVD) accounts for 15.6% of global disability-adjusted life years (DALYs) and imposes a substantial socioeconomic burden projected to exceed USD 1044 billion by 2030 [3]. Therefore, identifying key risk factors associated with CVD and mortality is of major public health importance. In this context, cardiovascular–kidney–metabolic (CKM) syndrome has recently been proposed as an integrated framework linking cardiovascular disease, kidney dysfunction, and metabolic risk factors [4]. CKM highlights the interaction among multi-organ risk factors and is classified into stages 0–4 based on organ involvement [5]. Notably, CKM stages 0–3 primarily reflect metabolic dysfunction and subclinical organ damage, which are potentially reversible and amenable to intervention [6]. Timely detection and appropriate management at these stages can help lower the likelihood of subsequent CVD events and death. This may also help ease the overall cardiovascular burden worldwide [7].

In people at CKM stages 0–3, metabolic abnormalities and functional decline may precede clinical cardiovascular events and progressively increase the likelihood of CVD, death, and other unfavorable outcomes [8]. Therefore, composite indices that integrate metabolic dysfunction and physiological reserve may provide a more comprehensive assessment of future cardiovascular and mortality risk. The triglyceride–glucose (TyG) index is regarded as a straightforward surrogate marker of insulin resistance, and higher values have been linked to increased risks of atherosclerosis, endothelial dysfunction, and cardiovascular events and death [9,10,11,12]. The frailty index (FI), reflecting cumulative health impairment and diminished physiological capacity, is widely used to predict poor outcomes in middle-aged and older populations [13,14] and has been consistently associated with cardiovascular events and hospitalization [15,16,17], as well as all-cause mortality [18,19]. Integrating TyG and FI into the TyG-FI index could provide a more comprehensive evaluation of cardiovascular risk.

Previous studies have reported that TyG and FI are useful for predicting cardiovascular disease and death [20,21,22], but most studies have relied on single measures. This limits a full understanding of cardiovascular risk. A composite approach integrating metabolic dysfunction and physiological reserve may therefore provide more comprehensive risk assessment [23]. However, evidence on the combined application of TyG and FI remains limited, particularly within the cardiovascular–kidney–metabolic framework and among individuals in stages 0–3, a potentially modifiable phase. More research is still needed to further clarify and assess the potential value of the TyG-FI index in forecasting incident CVD events and all-cause mortality in this group.

Building on the findings of Zhao et al. [24], who evaluated the association between TyG-FI and CVD and stroke in a prospective cohort study, this study further applies TyG-FI to the American Heart Association (AHA)-defined CKM stages 0–3 framework, conducting analyses based on the CHARLS and HRS longitudinal cohorts. We further evaluated the association between TyG-FI and the composite endpoint of new-onset CVD and all-cause mortality, as well as potential nonlinear patterns and stage-specific discriminatory ability within the CKM framework, to determine whether TyG-FI can provide exploratory risk stratification information at potentially intervenable CKM stages.

## 2. Materials and Methods

### 2.1. Study Design

**Figure 1:**
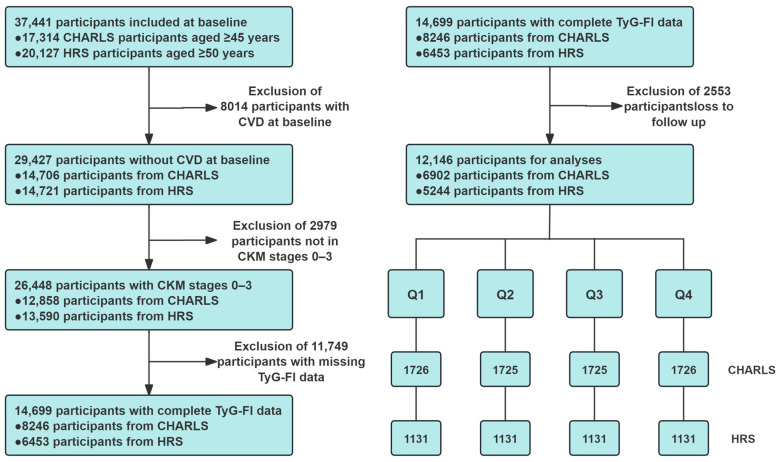
Participant selection process.

CHARLS included adults aged 45 and older in China, while HRS included adults aged 50 and older in the United States. Baseline data were taken from CHARLS wave 1 (2011) and HRS wave 13 (2016). Follow-up continued through CHARLS wave 5 (2020) and HRS wave 16 (2022). Both studies were reviewed and approved by the ethics committees of Peking University and the University of Michigan. Figure 1 shows the process of participant selection for both cohorts.

### 2.2. Data Collection

Data collected in this study encompassed four domains: sociodemographic characteristics, health-related conditions, anthropometric measurements, and laboratory parameters. Sociodemographic factors included age, sex, education, and marital status. Health-related conditions covered major chronic diseases and lifestyle behaviors, including hypertension, diabetes, dyslipidemia, chronic kidney disease, metabolic syndrome, smoking, alcohol consumption, and physical activity. Anthropometric and biochemical data were also collected, comprising body mass index, blood pressure, waist circumference, lipid profile, fasting glucose, HbA1c, and serum creatinine.

### 2.3. Definitions

CKM stages were determined following the AHA guidelines [5]. Detailed definitions are shown in Supplementary Table S1. The TyG was derived as ln[(fasting blood glucose (FBG, mg/dL) multiplied by triglycerides (TG, mg/dL)) divided by 2] [25]. The frailty index (FI) was constructed according to the standardized deficit accumulation approach proposed by Searle et al., using 30 health deficits in CHARLS and 28 in HRS (Supplementary Table S2) [26]. Deficits were selected based on established FI construction criteria and covered multiple health domains, including chronic conditions, functional limitations, cognitive function, and psychological well-being. Comparable deficits available in both cohorts were identified and harmonized according to their conceptual definitions. Each deficit was coded on a scale from 0 to 1, and the FI was calculated as the proportion of deficits present among the total number of eligible deficits. Consequently, the FI ranged from 0 to 1 in both cohorts, with higher scores indicating greater frailty [27]. The TyG-FI index was obtained by multiplying TyG by FI [24].

### 2.4. Outcome Ascertainment

In both the CHARLS and HRS cohorts, the primary outcome measure was the time to the composite endpoint of first cardiovascular event and all-cause mortality. This endpoint was selected because CKM syndrome reflects interrelated metabolic, renal, and cardiovascular abnormalities, which collectively increase the risk of cardiovascular disease incidence and mortality [5]. Similar methods have been used in cardiometabolic epidemiological studies to assess the association between the burden of comprehensive cardiometabolic risk factors and the incidence of CVD and all-cause mortality [28]. However, because the components of the composite endpoint may differ in clinical significance, assessment methods, or effect size, the results should be interpreted with caution, and sensitivity analyses should be conducted for specific endpoints [29,30]. Cardiovascular events included conditions participants reported as diagnosed by a doctor, such as heart disease or stroke. All-cause mortality was identified using information from the exit questionnaire, with death status reported by family members or proxies. Follow-up for the composite outcome began in 2013 for CHARLS and 2018 for HRS and continued until the first occurrence of the outcome.

### 2.5. Statistical Analyses

Participants were categorized by TyG-FI quartiles, and TyG-FI was also analyzed as a continuous variable. Descriptive statistics for continuous variables were reported as mean values with standard deviations, whereas categorical data were presented as frequencies and percentages. Between-group differences were evaluated using either Student’s *t*-test for continuous variables or the chi-square test for categorical variables, as appropriate. Kaplan–Meier curves with log-rank tests were used to compare cumulative incidence. Cox models were utilized to examine the relationship between TyG-FI and the outcome, and the findings were expressed as hazard ratios (HRs) together with 95% confidence intervals (CIs). Covariates were selected based on collinearity diagnostics (VIF < 5; Supplementary Table S3) and prior literature [31,32]. Nonlinear associations were assessed using restricted cubic splines, with piecewise Cox models applied as appropriate. Stratified analyses by age, sex, smoking, alcohol use, and CKM stage were performed and presented in forest plots. The discriminatory ability of TyG-FI for composite outcomes was evaluated using time-dependent ROC curves and compared head-to-head with the TyG, the FI, TyG-derived measures, and the Framingham Risk Score within the same cohort, using the same outcome definitions and analytical framework. To further evaluate whether TyG-FI can provide additional risk stratification information beyond existing risk data, this study conducted an incremental predictive value analysis using the net reclassification improvement index (NRI) and the integrated discrimination improvement index (IDI).

A series of supplementary analyses were carried out to assess the stability and robustness of the main findings: (1) repeating the analysis using CVD and all-cause mortality as separate outcomes (Supplementary Tables S7 and S8); (2) using competing risk models to consider death as a competing event (Supplementary Table S9); (3) using logistic regression to verify robustness (Supplementary Table S10); (4) removing participants with missing covariate information (Supplementary Table S11); (5) excluding CKM stage 0 participants (Supplementary Table S12); (6) reconstructing the FI after removing hypertension, diabetes, and arthritis (Supplementary Table S13); (7) excluding participants who experienced the outcome within the first year to reduce possible reverse causation (Supplementary Table S14); (8) conducting mediation analyses to assess whether hypertension, diabetes, chronic kidney disease, and metabolic syndrome mediated the association between TyG-FI and the outcomes (Supplementary Table S15); and (9) repeating the main models after excluding variables that may lie on the causal pathway, including HbA1c, total cholesterol, hypertension, diabetes, chronic kidney disease, and metabolic syndrome (Supplementary Table S16).

Missing covariate data were handled using multiple imputation by chained equations (MICE). Ten imputed datasets were generated and combined using Rubin’s rules. Detailed information on missing data is provided in Supplementary Table S6. Statistical analyses were conducted using R (version 4.5.1), and statistical significance was defined as a two-sided *p* < 0.05.

## 3. Results

### 3.1. Baseline Characteristics

A total of 6902 individuals in the CHARLS cohort (average age 59.55 years; 51.8% women) and 5244 individuals in the HRS cohort (average age 68.16 years; 61.1% women) were included in the study. In the CHARLS cohort, a total of 2696 participants experienced a composite outcome (first occurrence of incident CVD or all-cause death), among whom 1895 (70.4%) had incident CVD as the first event, and 801 (29.6%) had all-cause death as the first event. In the HRS cohort, 1526 participants experienced a composite outcome, including 886 (58.1%) with incident CVD as the first event and 640 (41.9%) with all-cause death as the first event. Participants were divided into four groups (Q1–Q4) based on TyG-FI levels. Across both cohorts, compared with those in the lowest quartile of TyG-FI, individuals in higher quartiles were generally older, tended to live alone, and were more likely to engage in light-intensity physical activity. They also showed a greater prevalence of hypertension, diabetes, dyslipidemia, CKD, and metabolic syndrome. However, in the CHARLS cohort, smoking decreased with higher TyG-FI levels, while the opposite trend was seen in HRS. Regarding body and lab measurements, participants in higher TyG-FI quartiles generally had larger waist circumference, higher systolic blood pressure, HbA1c, and fasting glucose, but lower HDL cholesterol than those in lower quartiles (Supplementary Table S4). Detailed characteristics of the unimputed data are shown in Supplementary Table S5.

### 3.2. Association of TyG-FI with Incident Outcome in CKM Stages 0–3

Follow-up averaged 6.9 years in the CHARLS cohort and 4.6 years in the HRS cohort, during which 2696 (39.1%) and 1526 (29.1%) participants, respectively, experienced the outcome. Kaplan–Meier curves of cumulative incidence (Supplementary Figure S1) showed a stepwise increase in outcome incidence across increasing quartiles of TyG-FI (Q1–Q4) in both cohorts, with the highest incidence observed in Q4; log-rank tests indicated significant differences among groups (all *p* < 0.001). Cox regression revealed a clear positive association between TyG-FI and new outcomes among individuals classified into CKM stages 0–3. When treated as a continuous variable, a 1-unit increase in TyG-FI was associated with a 38.4% higher risk of the outcome in the unadjusted model, and this relationship remained significant after stepwise adjustment (adjusted HR = 1.253, 95% CI: 1.213–1.294). When treated as a categorical variable, participants in the CHARLS cohort showed progressively higher risk across TyG-FI quartiles compared with Q1 in the multivariable model with full adjustment for covariates (HRs for Q2, Q3, and Q4: 1.510, 1.544, and 2.202). Similar patterns were found in the HRS cohort, where a 1-unit increase in TyG-FI corresponded to a 24.0% higher risk in model 3, and the hazard ratios for Q2, Q3, and Q4 were 1.516, 1.874, and 2.601, respectively. See Table 1. The results using CVD and all-cause mortality as separate outcomes were consistent with the main analysis, showing similar directions of association (Supplementary Tables S6 and S7).

**Figure 2:**
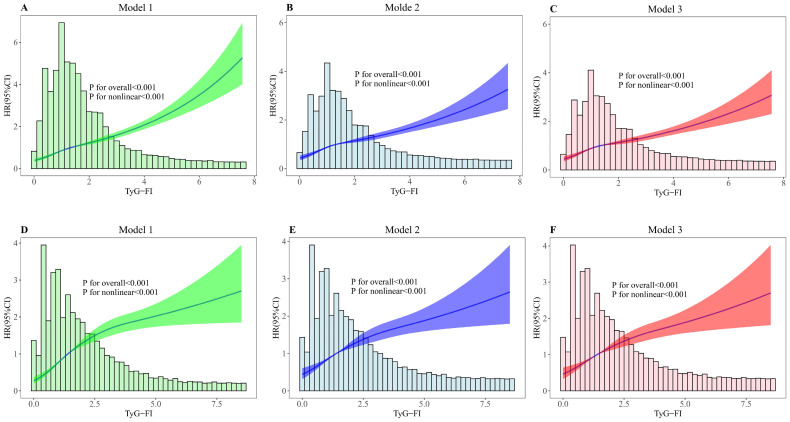
Nonlinear relationship between TyG-FI and outcome. (**A**) Model 1 in CHARLS; (**B**) Model 2 in CHARLS; (**C**) Model 3 in CHARLS; (**D**) Model 1 in HRS; (**E**) Model 2 in HRS; (**F**) Model 3 in HRS. The solid lines represent the estimated hazard ratios (HRs), and the shaded areas represent the 95% confidence intervals (95% CIs). Histograms show the distribution of TyG-FI values.

To further examine whether TyG-FI was linked to the outcome in a linear or nonlinear manner, RCS analyses were performed. In both cohorts, a notable nonlinear association between TyG-FI and outcome was observed in individuals classified as CKM stages 0–3 (overall *p* < 0.001; nonlinearity *p* < 0.001; Figure 2). Threshold effect analyses using a two-segment Cox regression identified an inflection point at 1.01 in the CHARLS cohort. Below this cutoff value, TyG-FI was strongly associated with incident outcome (HR = 2.23, 95% CI: 1.65–3.01; *p* < 0.001), whereas above the threshold, the relationship was still statistically significant, although its strength was reduced (HR = 1.21, 95% CI: 1.17–1.26; *p* < 0.001). In the HRS cohort, an inflection point was identified at 2.29. TyG-FI was significantly linked to increased outcome risk below this threshold (HR = 1.54, 95% CI: 1.37–1.73; *p* < 0.001); the association was weaker but remained statistically significant beyond this cutoff (HR = 1.11, 95% CI: 1.04–1.19; *p* = 0.002). Likelihood ratio tests in both cohorts confirmed a threshold effect (all *p* < 0.001; Table 2).

### 3.3. Subgroup Analyses

**Figure 3:**
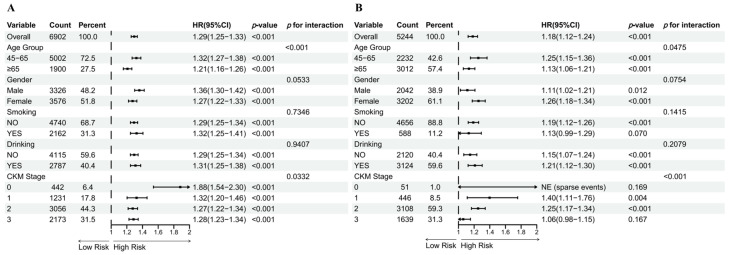
Subgroup and interaction analyses of TyG-FI in CHARLS (**A**) and HRS (**B**).

Subgroup analyses were performed by age, sex, smoking status, alcohol consumption, and CKM stage (Figure 3). Higher TyG-FI was generally associated with a greater risk across most subgroups in both cohorts (*p* < 0.05). In both cohorts, significant interactions were observed between TyG-FI and both age and CKM stage (P for interaction < 0.05). TyG-FI showed a clear association with the composite outcome across all stages in the CHARLS cohort (all *p* < 0.001), with the strongest association at CKM stage 0. In the HRS cohort, clear associations appeared in CKM stages 1–2, whereas the association did not reach statistical significance in CKM stage 3.

### 3.4. Discriminatory Performance of TyG-FI for Incident Outcome

**Figure 4:**
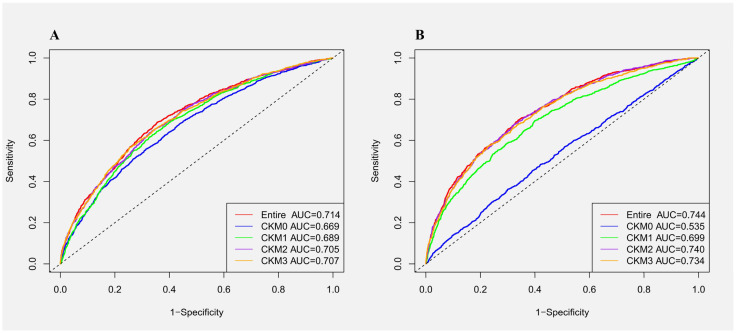
ROC curves of TyG-FI for incident outcome in CHARLS (**A**) and HRS (**B**). The dashed diagonal line indicates a classifier with no discriminative ability (AUC = 0.5).

Time-dependent receiver operating characteristic (ROC) curves were used to evaluate the discriminative ability of TyG-FI for incident composite outcomes among participants in CKM stages 0–3 (Figure 4). In the CHARLS cohort, the overall area under the curve (AUC) of TyG-FI was 0.714, and the stage-specific AUCs for CKM stages 0–3 were 0.669, 0.689, 0.705, and 0.707, respectively. In the HRS cohort, the overall AUC of TyG-FI was 0.744, with corresponding AUCs of 0.535, 0.699, 0.740, and 0.734 for CKM stages 0, 1, 2, and 3, respectively. These findings indicate that TyG-FI demonstrated moderate overall discriminative performance for the composite outcome rather than strong predictive ability. Head-to-head comparisons with the TyG index, FI, TyG-BMI, TyG-BRI, TyG-WWI, TyG-CVAI, and the Framingham Risk Score are presented in Supplementary Figure S2. In the CHARLS cohort, the IDI was 0.021 (95% CI: 0.014–0.028) and the continuous NRI was 0.119 (95% CI: 0.078–0.149) (both *p* < 0.001). Corresponding values in the HRS cohort were 0.019 (95% CI: 0.008–0.027) and 0.148 (95% CI: 0.107–0.185), respectively (both *p* < 0.001). See Supplementary Table S17.

## 4. Discussion

In this study, based on two large longitudinal cohorts (CHARLS and HRS), we first evaluated the general association between TyG-FI and adverse cardiovascular outcomes, and further positioned TyG-FI within the AHA-defined CKM stages 0–3 framework, with a focus on its value for risk stratification in individuals who have not yet developed clinical CVD and thus remain within a potentially actionable intervention window. The results showed that higher TyG-FI was consistently associated with an increased risk of the composite outcome of incident CVD and all-cause mortality in both cohorts. These associations remained robust after sequential adjustment for demographic characteristics, lifestyle factors, metabolic markers, and CKM-related comorbidities. More importantly, restricted cubic spline analyses and piecewise Cox regression models indicated that the relationship between TyG-FI and outcomes was not linear but exhibited discernible threshold patterns in both cohorts. In addition, subgroup analyses indicated that the strength of the association varied by age and CKM stage, collectively suggesting a stage-dependent role of TyG-FI in early to mid-stage CKM populations. Time-dependent ROC analyses further demonstrated that TyG-FI had moderate discriminative ability for incident composite outcomes. The generally consistent findings across the CHARLS and HRS cohorts suggest that TyG-FI may provide exploratory risk stratification information in individuals at CKM stages 0–3; however, current evidence is insufficient to support its use as an independent clinical prediction tool.

From a biological perspective, the triglyceride-glucose (TyG) index, as a simple surrogate marker of insulin resistance, is closely associated with the progression of atherosclerosis, endothelial dysfunction, chronic low-grade inflammation, dyslipidemia, and an increased risk of cardiovascular events and mortality [33,34,35,36]. At the same time, the frailty index (FI), which reflects multisystem functional decline and reduced physiological reserve through the accumulation of health deficits, has also been well established as being associated with cardiovascular events, hospitalization, all-cause mortality, and other adverse health outcomes [37,38,39,40]. More importantly, insulin resistance and frailty are not two entirely independent risk dimensions. Long-term insulin resistance may be linked to subsequent frailty status, frailty progression, and increased cardiovascular risk, while frailty itself may further amplify cardiovascular risk through mechanisms such as increased inflammatory burden, sarcopenia, disruption of metabolic homeostasis, and neuroendocrine and immune dysregulation [41,42,43,44]. Therefore, integrating TyG with FI within the CKM staging framework allows simultaneous capture of both metabolic dysregulation and reduced physiological reserve—two key risk dimensions closely related to CKM progression. Unlike existing TyG-derived indices that primarily emphasize insulin resistance and adiposity-related features, the TyG-FI further incorporates information on functional decline and biological vulnerability, thereby better aligning with the concept of multidimensional, continuous risk accumulation in CKM syndrome [31,45].

In individuals with CKM syndrome stages 0–3, the TyG-FI index was consistently associated with higher hazards of outcomes. This association remained robust when TyG-FI was examined as either a continuous or grouped variable, and after stepwise adjustment for demographic, clinical, and laboratory factors. This aligns with prior studies in the general population [24], which reported that elevated TyG is associated with greater cardiovascular risk, reflecting underlying metabolic abnormalities [20]. Recent studies indicate the combined effects of metabolic dysfunction and frailty in exacerbating cardiovascular disease burden [23]. By integrating TyG with frailty status, TyG-FI may better capture combined metabolic disturbance and functional decline. Although individuals with CKM syndrome typically have a higher baseline risk due to multiple metabolic and organ dysfunctions, potentially attenuating the marginal effect of further risk elevation [46], the consistent associations across both cohorts support TyG-FI as an independent factor associated with incident CVD and mortality for heart events and death in this group. Notably, the association between TyG-FI and incident CVD was slightly stronger than that observed for the composite endpoint. This finding suggests that the inclusion of non-cardiovascular deaths may dilute the effect estimates, as such deaths may not share the same underlying biological pathways related to insulin resistance and frailty. From a clinical perspective, TyG-FI may be better understood as a simple, readily obtainable exploratory marker that could help identify individuals with a higher cardiometabolic burden within the CKM spectrum. Given its moderate AUC performance and the relatively limited incremental improvement over certain reference indicators, it is currently more appropriately considered a useful complement to existing risk assessment systems, providing additional information for risk stratification rather than serving as an independent clinical decision-making tool or a substitute for established risk prediction scores.

Another important extension of this study is the systematic evaluation of the nonlinear relationship and potential threshold effects between TyG-FI and outcomes, rather than assuming a uniform increase in risk with higher levels of the index. Restricted cubic spline analyses and piecewise Cox models identified different inflection points in the CHARLS and HRS cohorts, suggesting that the effect of TyG-FI on outcome risk may be jointly influenced by baseline risk profiles, age distribution, CKM stage composition, and cohort characteristics. Below the inflection point, increases in TyG-FI may primarily reflect the combined accumulation of early insulin resistance, mild frailty, and low-grade inflammation, during which endothelial function, metabolic homeostasis, and physiological reserve may still retain a certain degree of reversibility; accordingly, each unit increase in TyG-FI is associated with a more pronounced change in risk [47,48]. In contrast, beyond a certain threshold, individuals may already have more substantial multisystem dysfunction, lipotoxic injury, oxidative stress, and vascular structural changes, and the marginal contribution of further increases in TyG-FI to risk discrimination may diminish, resulting in a flattening of the risk slope [49,50]. Taken together, these results indicate that TyG-FI is more sensitive to cardiovascular risk at low-to-moderate levels, underscoring the possible practical and preventive benefit of prompt identification and intervention before risk exceeds critical thresholds.

Subgroup analyses showed a link between TyG-FI and higher chances of incident outcomes across different demographic strata. Notably, interactions with age and CKM stage appeared, with the association stronger in participants aged 45–65 and in early CKM stages. Stratified analyses indicated that TyG-FI’s link with the outcome was clearer in the early CKM stages, consistent with previous studies [51]. This may suggest that, in groups with lower baseline risk, such as individuals with CKM stages 0–2, TyG-FI may help identify those likely to benefit from early lifestyle modification to prevent progression to more advanced CKM stages.

Time-dependent ROC analysis showed AUCs of 0.714 in CHARLS and 0.744 in HRS, with a gradual increase in discriminative ability across advancing CKM stages in both cohorts. These findings suggest that TyG-FI may be more informative for identifying high-risk individuals in advanced CKM stages, whereas its predictive performance remains moderate in earlier stages. Overall, the clinical utility of TyG-FI in risk stratification appears to vary with CKM severity, potentially providing more reliable information in populations with higher baseline risk. In individuals with CKM stages 0–3, TyG-FI may serve as an exploratory adjunctive marker for risk stratification, complementing established risk assessment approaches. In the same cohorts, comparative analyses were conducted against previously reported TyG-derived indices (TyG-BMI, TyG-BRI, TyG-WWI, and TyG-CVAI), as well as the TyG index, FI, and the Framingham risk score [31]. In these head-to-head comparisons, TyG-FI showed relatively higher AUC values, although the differences among indices were modest. Incremental predictive analyses further indicated that adding TyG-FI to the model improved both discrimination and individual-level risk reclassification. In the CHARLS and HRS cohorts, the addition of TyG-FI increased the average predicted probability separation between event and non-event groups by approximately 2.0% and 1.9%, respectively. Moreover, approximately 11.9% and 14.8% of individuals were more appropriately reclassified in risk strata. Overall, both IDI and NRI analyses consistently support a modest but meaningful incremental predictive value of TyG-FI, although the magnitude of improvement remains limited.

Nevertheless, some limitations of this study need to be noted. First, as in prior research, self- or physician-reported outcomes and covariates may have underestimated the incidence and limited subtype classification, potentially affecting the accuracy of the results. Second, despite the prospective design and adjustment for confounders, the observational design prevents causal conclusions and cannot rule out residual confounding. Third, baseline-only measurements of TyG-FI and covariates may not capture longitudinal changes, possibly affecting the accuracy of their associations with outcome risk. Fourth, since CHARLS and HRS primarily include adults aged 45 years and older and 50 years and older, respectively, the findings of this study should be interpreted as applicable mainly to middle-aged and older adults within CKM stages 0–3, and should not be directly extrapolated to younger populations. The frailty index reflects cumulative health deficits and declining physiological reserve, and is strongly age-dependent. In younger individuals, the burden of frailty, the incidence of short-term outcomes, and the pattern of early CKM risk may differ substantially. Therefore, the exploratory TyG-FI thresholds identified in this study require further validation in cohorts with a broader age range and repeated longitudinal measurements. Finally, mediation analyses showed minimal or no mediation through major CKM-related diseases, and the associations remained robust after excluding overlapping disease-related frailty items and relevant covariates. Nevertheless, because the TyG-FI incorporates both metabolic and frailty-related components, and some frailty deficits may be correlated with CKM-related conditions, residual conceptual overlap cannot be entirely ruled out.

## 5. Conclusions

In participants with CKM stages 0–3 in the CHARLS and HRS cohorts, higher TyG-FI was associated with an increased risk of the composite outcome of incident CVD and all-cause mortality, with evidence of a nonlinear relationship. TyG-FI may serve as an exploratory risk stratification marker in middle-aged and older adults with early-to-mid-stage CKM. However, given the observational study design, the reliance of some outcomes on self-reported information, and the restriction of the study population to adults aged ≥45/50 years, its clinical applicability requires further validation.

## Figures and Tables

**Table 1:** Association of TyG-FI with outcome risk across CKM stages 0–3.

	Model 1		Model 2		Model 3	
	HR (95% CI)	*p* Value	HR (95% CI)	*p* Value	HR (95% CI)	*p* Value
CHARLS						
TyG-FI	1.384 (1.345–1.424)	<0.001	1.270 (1.231–1.310)	<0.001	1.253 (1.213–1.294)	<0.001
TyG-FI (Q1–Q4)						
Q1	Reference		Reference		Reference	
Q2	1.610 (1.424–1.821)	<0.001	1.533 (1.355–1.735)	<0.001	1.510 (1.334–1.709)	<0.001
Q3	1.770 (1.567–1.998)	<0.001	1.597 (1.412–1.807)	<0.001	1.544 (1.363–1.749)	<0.001
Q4	2.923 (2.607–3.276)	<0.001	2.308 (2.046–2.602)	<0.001	2.202 (1.949–2.489)	<0.001
HRS						
TyG-FI	1.336 (1.296–1.378)	<0.001	1.248 (1.202–1.296)	<0.001	1.240 (1.192–1.291)	<0.001
TyG-FI (Q1–Q4)						
Q1	Reference		Reference		Reference	
Q2	1.906 (1.590–2.285)	<0.001	1.554 (1.293–1.868)	<0.001	1.516 (1.258–1.828)	<0.001
Q3	2.702 (2.273–3.212)	<0.001	1.947 (1.624–2.335)	<0.001	1.874 (1.554–2.260)	<0.001
Q4	3.995 (3.382–4.719)	<0.001	2.706 (2.251–3.252)	<0.001	2.601 (2.144–3.154)	<0.001

Model 1: unadjusted; Model 2: adjusted for age, gender, education, marital status, smoking, drinking, physical activity, WC, BMI; Model 3: adjusted for age, gender, education, marital status, smoking, drinking, physical activity, WC, SBP, DBP, BMI, TC, HbA1c, HDL, LDL, Cr, HTN, DM, CKD, Mets.

**Table 2:** Threshold effect of TyG-FI on incident outcome using a two-piecewise Cox model.

	Adjusted HR (95% CI)	*p* Value
CHARLS		
Total	1.27 (1.23–1.31)	<0.001
Inflection point	1.01	
<1.01	2.23 (1.65–3.01)	<0.001
≥1.00	1.21 (1.17–1.26)	<0.001
P for Log-likelihood ratio		<0.001
HRS		
Total	1.25 (1.20–1.3)	<0.001
Inflection point	2.29	
<2.29	1.54 (1.37–1.73)	<0.001
≥2.29	1.11 (1.04–1.19)	0.002
P for Log-likelihood ratio		<0.001

## Data Availability

Data supporting the findings of this study are publicly available from the official websites of the CHARLS (http://charls.pku.edu.cn, accessed on 17 March 2026) and the HRS (https://hrs.isr.umich.edu, accessed on 24 March 2026).

## Supplementary Materials

The following supporting information can be downloaded at: https://www.mdpi.com/article/10.3390/metabo16060426/s1, Supplementary Table S1: Staging of CKM syndrome from stages 0 to 3; Supplementary Table S2: Detailed description of the 30 indicators used to construct the frailty index in CHARLS and HRS, along with their corresponding cutoff values; Supplementary Table S3: Collinearity Statistics; Supplementary Table S4: Baseline Characteristics of Participants in the CHARLS and HRS; Supplementary Table S5: Unimputed Baseline Characteristics of Participants; Supplementary Table S6: Missing data for study variables in CHARLS and HRS cohorts; Supplementary Table S7: Association Between the TyG-FI Index and the Risk of Cardiovascular Disease Among Individuals With CKM Stages 0–3; Supplementary Table S8: Association Between the TyG-FI Index and the Risk of all-cause mortality Among Individuals With CKM Stages 0–3; Supplementary Table S9: Association Between TyG-FI and Incident Composite Cardiovascular and All-Cause Mortality Outcomes in CKM Stages 0–3 (Competing Risk Model); Supplementary Table S10: Association Between TyG-FI and Incident Composite Cardiovascular and All-Cause Mortality Outcomes in CKM Stages 0–3 (Logistic regression model); Supplementary Table S11: Association Between TyG-FI and Incident Composite Cardiovascular and All-Cause Mortality Outcomes in CKM Stages 0–3 (Exclude individuals with missing values); Supplementary Table S12: Association Between TyG-FI and Incident Composite Cardiovascular and All-Cause Mortality Outcomes in CKM Stages 1–3; Supplementary Table S13: Association Between TyG-FI and Incident Composite Cardiovascular and All-Cause Mortality Outcomes in CKM Stages 0–3 (Reconstruction of the Frailty Index); Supplementary Table S14: Association Between TyG-FI and Incident Composite Cardiovascular and All-Cause Mortality Outcomes in CKM Stages 0–3 (Excluded events within 1 year); Supplementary Table S15: Mediation analyses of potential cardiometabolic mediators in the association between TyG-FI and outcome; Supplementary Table S16: Association Between TyG-FI and Incident Composite Cardiovascular and All-Cause Mortality Outcomes in CKM Stages 0–3 (excluded potential mediators); Supplementary Table S17: Incremental predictive value of the new model assessed by IDI and NRI in CHARLS and HRS cohorts; Supplementary Figure S1: Kaplan–Meier curves by TyG–FI quartiles in CHARLS (A) and HRS (B); Supplementary Figure S2. Comparison of ROC curves for TyG-FI and other risk indicators in CHARLS (A) and HRS (B).

## Abbreviations

The following abbreviations are used in this manuscript:

CHARLS	China Health and Retirement Longitudinal Study
CI	Confidence interval
CKM	cardiovascular-kidney-metabolic syndrome
CVD	Cardiovascular disease
FI	Frailty index
HR	Hazard ratio
HRS	Health and Retirement Study
RCS	Restricted Cubic Spline
TDROC	Time-dependent Receiver Operating Characteristic
TyG	Triglyceride–Glucose index

## References

[1] (2025). Global Burden of Cardiovascular Diseases and Risks 2023 Collaborators. Global, Regional, and National Burden of Cardiovascular Diseases and Risk Factors in 204 Countries and Territories, 1990–2023. J. Am. Coll. Cardiol..

[2] Vos T., Lim S.S., Abbafati C., Abbas K.M., Abbasi M., Abbasifard M., Abbasi-Kangevari M., Abbastabar H., Abd-Allah F., Abdelalim A. (2020). Global Burden of 369 Diseases and Injuries in 204 Countries and Territories, 1990–2019: A Systematic Analysis for the Global Burden of Disease Study 2019. Lancet.

[3] Bloom D.E., Cafiero E., Jané-Llopis E., Abrahams-Gessel S., Bloom L.R., Fathima S., Feigl A.B., Gaziano T., Hamandi A., Mowafi M. (2012). The Global Economic Burden of Noncommunicable Diseases.

[4] Mutruc V., Bologa C., Șorodoc V., Ceasovschih A., Morărașu B.C., Șorodoc L., Catar O.E., Lionte C. (2025). cardiovascular-kidney-metabolic Syndrome: A New Paradigm in Clinical Medicine or Going Back to Basics?. J. Clin. Med..

[5] Ndumele C.E., Neeland I.J., Tuttle K.R., Chow S.L., Mathew R.O., Khan S.S., Coresh J., Baker-Smith C.M., Carnethon M.R., Després J.-P. (2023). A Synopsis of the Evidence for the Science and Clinical Management of Cardiovascular-Kidney-Metabolic (CKM) Syndrome: A Scientific Statement from the American Heart Association. Circulation.

[6] Gunnarsson S., Vito O., Unwin R.J. (2026). Cardiovascular-Kidney-Metabolic Syndrome: Prevalence, Risks, Disease Trajectories, and Early-Stage Management. Am. J. Physiol. Cell Physiol..

[7] Stewart J., Manmathan G., Wilkinson P. (2017). Primary Prevention of Cardiovascular Disease: A Review of Contemporary Guidance and Literature. JRSM Cardiovasc. Dis..

[8] Wang Z., Chen J., Zhu L., Jiao S., Chen Y., Sun Y. (2023). Metabolic Disorders and Risk of Cardiovascular Diseases: A Two-Sample Mendelian Randomization Study. BMC Cardiovasc. Disord..

[9] Hong S., Han K., Park C.-Y. (2020). The Triglyceride Glucose Index Is a Simple and Low-Cost Marker Associated with Atherosclerotic Cardiovascular Disease: A Population-Based Study. BMC Med..

[10] Li Y., Yi M., Wang X., Zhang Y., Xiao K., Si J., Sun L., Zhang H., Sun J., Liu Z. (2024). Association between Triglyceride-Glucose Index and Endothelial Dysfunction. Endocrine.

[11] Fu B., Zeng Y., Wang M., Zhao L., Sun L., Wang T., Dong J., Yang W., Hua W. (2024). The Triglyceride-Glucose Index Is a Predictor of Major Adverse Cardiovascular Events in Patients with Coronary Artery Disease and Psoriasis: A Retrospective Cohort Study. Diabetol. Metab. Syndr..

[12] Moon J.H., Kim Y., Oh T.J., Moon J.H., Kwak S.H., Park K.S., Jang H.C., Choi S.H., Cho N.H. (2023). Triglyceride-Glucose Index Predicts Future Atherosclerotic Cardiovascular Diseases: A 16-Year Follow-up in a Prospective, Community-Dwelling Cohort Study. Endocrinol. Metab..

[13] Kaskirbayeva D., West R., Jaafari H., King N., Howdon D., Shuweihdi F., Clegg A., Nikolova S. (2023). Progression of Frailty as Measured by a Cumulative Deficit Index: A Systematic Review. Ageing Res. Rev..

[14] Walston J.D., Bandeen-Roche K. (2015). Frailty: A Tale of Two Concepts. BMC Med..

[15] Zhou Q., Wang Z., Xu Q., Xia X., Gao M., Zhang L., Tian X., He D., Wang A. (2026). Physical Function Frailty Trajectory before and after Cardiovascular Event among Older Adults in Three Multinational Cohorts. BMC Public Health.

[16] Uchmanowicz I., Lee C.S., Vitale C., Manulik S., Denfeld Q.E., Uchmanowicz B., Rosińczuk J., Drozd M., Jaroch J., Jankowska E.A. (2020). Frailty and the Risk of All-Cause Mortality and Hospitalization in Chronic Heart Failure: A Meta-Analysis. ESC Heart Fail..

[17] Ekram A.R.M.S., Tonkin A.M., Ryan J., Beilin L., Ernst M.E., Espinoza S.E., McNeil J.J., Nelson M.R., Reid C.M., Newman A.B. (2023). The Association between Frailty and Incident Cardiovascular Disease Events in Community-Dwelling Healthy Older Adults. Am. Heart J. Plus Cardiol. Res. Pract..

[18] Veronese N., Cereda E., Stubbs B., Solmi M., Luchini C., Manzato E., Sergi G., Manu P., Harris T., Fontana L. (2017). Risk of Cardiovascular Disease Morbidity and Mortality in Frail and Pre-Frail Older Adults: Results from a Meta-Analysis and Exploratory Meta-Regression Analysis. Ageing Res. Rev..

[19] Crow R.S., Lohman M.C., Titus A.J., Bruce M.L., Mackenzie T.A., Bartels S.J., Batsis J.A. (2018). Mortality Risk Along the Frailty Spectrum: Data from the National Health and Nutrition Examination Survey 1999 to 2004. J. Am. Geriatr. Soc..

[20] Liu X., Tan Z., Huang Y., Zhao H., Liu M., Yu P., Ma J., Zhao Y., Zhu W., Wang J. (2022). Relationship between the Triglyceride-Glucose Index and Risk of Cardiovascular Diseases and Mortality in the General Population: A Systematic Review and Meta-Analysis. Cardiovasc. Diabetol..

[21] Kojima G., Iliffe S., Walters K. (2018). Frailty Index as a Predictor of Mortality: A Systematic Review and Meta-Analysis. Age Ageing.

[22] Wang H., Fu Q., Xiao S., Ma X., Liao Y., Kang C., Yang R. (2024). Predictive Value of the Triglyceride-Glucose Index for Short- and Long-Term All-Cause Mortality in Patients with Critical Coronary Artery Disease: A Cohort Study from the MIMIC-IV Database. Lipids Health Dis..

[23] Liu M., Li J., Yan K., Zhang K., Wang M., Guo J., Heisha N., Yang Y., Yuan J., Ye Y. (2025). Synergistic Interaction and Cumulative Effect between Frailty and Triglyceride-Glucose Index Exacerbate Cardiovascular Disease Burden in Middle-Aged and Older Adults. Diabetes Res. Clin. Pract..

[24] Zhao Y.-C., Wu S.-Q., Li J.-K., Sun Z.-H., Zhang B.-K., Fu R., Yan M. (2025). Predictive Value of the Combined Triglyceride-Glucose and Frailty Index for Cardiovascular Disease and Stroke in Two Prospective Cohorts. Cardiovasc. Diabetol..

[25] Guerrero-Romero F., Simental-Mendía L.E., González-Ortiz M., Martínez-Abundis E., Ramos-Zavala M.G., Hernández-González S.O., Jacques-Camarena O., Rodríguez-Morán M. (2010). The Product of Triglycerides and Glucose, a Simple Measure of Insulin Sensitivity. Comparison with the Euglycemic-Hyperinsulinemic Clamp. J. Clin. Endocrinol. Metab..

[26] Searle S.D., Mitnitski A., Gahbauer E.A., Gill T.M., Rockwood K. (2008). A Standard Procedure for Creating a Frailty Index. BMC Geriatr..

[27] Rockwood K., Mitnitski A. (2007). Frailty in Relation to the Accumulation of Deficits. J. Gerontol. Ser. A.

[28] Cao X., Zhang L., Wang X., Chen Z., Zheng C., Chen L., Zhou H., Cai J., Hu Z., Tian Y. (2023). Cardiovascular Disease and All-Cause Mortality Associated with Individual and Combined Cardiometabolic Risk Factors. BMC Public Health.

[29] Cordoba G., Schwartz L., Woloshin S., Bae H., Gøtzsche P.C. (2010). Definition, Reporting, and Interpretation of Composite Outcomes in Clinical Trials: Systematic Review. BMJ.

[30] Ferreira-González I., Busse J.W., Heels-Ansdell D., Montori V.M., Akl E.A., Bryant D.M., Alonso-Coello P., Alonso J., Worster A., Upadhye S. (2007). Problems with Use of Composite End Points in Cardiovascular Trials: Systematic Review of Randomised Controlled Trials. BMJ.

[31] Yue Y., Li P., Sun Z., Murayama R., Li Z., Hashimoto K., Zhang Y. (2025). Association of Novel Triglyceride-Glucose-Related Indices with Incident Stroke in Early-Stage Cardiovascular-Kidney-Metabolic Syndrome. Cardiovasc. Diabetol..

[32] Li F., Wang Y., Shi B., Sun S., Wang S., Pang S., Wu X. (2024). Association between the Cumulative Average Triglyceride Glucose-Body Mass Index and Cardiovascular Disease Incidence among the Middle-Aged and Older Population: A Prospective Nationwide Cohort Study in China. Cardiovasc. Diabetol..

[33] Wan Y., Zhang Z., Ling Y., Cui H., Tao Z., Pei J., Maimaiti A., Bai H., Wu Y., Li J. (2023). Association of Triglyceride-Glucose Index with Cardiovascular Disease among a General Population: A Prospective Cohort Study. Diabetol. Metab. Syndr..

[34] Li J., Dong Z., Wu H., Liu Y., Chen Y., Li S., Zhang Y., Qi X., Wei L. (2023). The Triglyceride-Glucose Index Is Associated with Atherosclerosis in Patients with Symptomatic Coronary Artery Disease, Regardless of Diabetes Mellitus and Hyperlipidaemia. Cardiovasc. Diabetol..

[35] Lin X., Xie S., Pu B., Wang Z., Zhang H., Li Y., Tang J., Yu B., Jiang S. (2025). Triglyceride–Glucose Index in the Associations between Chronic Low-Grade Inflammation and Carotid Intima-Media Thickness: Mediation and Effect-Modification across Triglyceride–Glucose Strata. BMC Cardiovasc. Disord..

[36] Li H., Jiang Y., Su X., Meng Z. (2023). The Triglyceride Glucose Index Was U-Shape Associated with All-Cause Mortality in Population with Cardiovascular Diseases. Diabetol. Metab. Syndr..

[37] Liu X., Dai G., He Q., Ma H., Hu H. (2022). Frailty Index and Cardiovascular Disease among Middle-Aged and Older Chinese Adults: A Nationally Representative Cross-Sectional and Follow-up Study. J. Cardiovasc. Dev. Dis..

[38] Ueno K., Ko T., Suzuki Y., Kaneko H., Kamiya K., Okada A., Fujiu K., Takeda N., Morita H., Node K. (2025). Frailty and Its Components and Cardiovascular Outcomes in Older Adults: A Nationwide Epidemiological Study. Geriatr. Gerontol. Int..

[39] Li X., Ploner A., Karlsson I.K., Liu X., Magnusson P.K.E., Pedersen N.L., Hägg S., Jylhävä J. (2019). The Frailty Index Is a Predictor of Cause-Specific Mortality Independent of Familial Effects from Midlife Onwards: A Large Cohort Study. BMC Med..

[40] Kim D.H., Rockwood K. (2024). Frailty in Older Adults. N. Engl. J. Med..

[41] Walston J., McBurnie M.A., Newman A., Tracy R.P., Kop W.J., Hirsch C.H., Gottdiener J., Fried L.P., Cardiovascular Health Study (2002). Frailty and Activation of the Inflammation and Coagulation Systems with and without Clinical Comorbidities: Results from the Cardiovascular Health Study. Arch. Intern. Med..

[42] Westbrook R., Zhang C., Yang H., Tian J., Guo S., Xue Q.-L., Walston J., Le A., Abadir P.M. (2022). Metabolomics-Based Identification of Metabolic Dysfunction in Frailty. J. Gerontol. Ser. A.

[43] Ng T.P., Lu Y., Choo R.W.M., Tan C.T.Y., Nyunt M.S.Z., Gao Q., Mok E.W.H., Larbi A. (2018). Dysregulated Homeostatic Pathways in Sarcopenia among Frail Older Adults. Aging Cell.

[44] Ke Z., Wen H., Huang R., Xu X., Yang K., Liu W., Wang S., Zhang X., Guo Y., Liao X. (2024). Long-Term Insulin Resistance Is Associated with Frailty, Frailty Progression, and Cardiovascular Disease. J. Cachexia Sarcopenia Muscle.

[45] Ndumele C.E., Rangaswami J., Chow S.L., Neeland I.J., Tuttle K.R., Khan S.S., Coresh J., Mathew R.O., Baker-Smith C.M., Carnethon M.R. (2023). Cardiovascular-Kidney-Metabolic Health: A Presidential Advisory from the American Heart Association. Circulation.

[46] Sebastian S.A., Padda I., Johal G. (2024). Cardiovascular-Kidney-Metabolic (CKM) Syndrome: A State-of-the-Art Review. Curr. Probl. Cardiol..

[47] Kosmas C.E., Bousvarou M.D., Kostara C.E., Papakonstantinou E.J., Salamou E., Guzman E. (2023). Insulin Resistance and Cardiovascular Disease. J. Int. Med. Res..

[48] de Rooij S.R., Nijpels G., Nilsson P.M., Nolan J.J., Gabriel R., Bobbioni-Harsch E., Mingrone G., Dekker J.M., Relationship Between Insulin Sensitivity and Cardiovascular Disease (RISC) Investigators (2009). Low-Grade Chronic Inflammation in the Relationship between Insulin Sensitivity and Cardiovascular Disease (RISC) Population: Associations with Insulin Resistance and Cardiometabolic Risk Profile. Diabetes Care.

[49] Vilas-Boas E.A., Almeida D.C., Roma L.P., Ortis F., Carpinelli A.R. (2021). Lipotoxicity and β-Cell Failure in Type 2 Diabetes: Oxidative Stress Linked to NADPH Oxidase and ER Stress. Cells.

[50] Johnson N., Qu J., Wagatsuma K., Su Y., Du B., He Y., Yang P. (2025). Frailty and Cardiovascular Disease: A Bidirectional Relationship with Clinical Implications. Front. Cardiovasc. Med..

[51] Kim B.S., Kim H.-J., Moon S., Kim H., Lee J., Shin J.-H. (2025). Association of the Triglyceride-Glucose Index with Cardiovascular Outcomes across Cardiovascular-Kidney-Metabolic Syndrome Stages. Korean J. Intern. Med..

